# Predictive and Prognostic Value of Ribonucleotide Reductase Regulatory Subunit M1 and Excision Repair Cross-Complementation Group 1 in Advanced Urothelial Carcinoma (UC) Treated with First-Line Gemcitabine Plus Platinum Combination Chemotherapy

**DOI:** 10.1371/journal.pone.0133371

**Published:** 2015-07-22

**Authors:** Miso Kim, Ja Hyeon Ku, Cheol Kwak, Hyeon Hoe Kim, Eunsik Lee, Bhumsuk Keam, Tae Min Kim, Dae Seog Heo, Se-Hoon Lee, Kyung Chul Moon

**Affiliations:** 1 Division of Hematology/Oncology, Department of Internal Medicine, Seoul National University Hospital, Seoul, Korea; 2 Department of Urology, Seoul National University Hospital, Seoul, Korea; 3 Cancer Research Institute, Seoul National University College of Medicine, Seoul, Korea; 4 Division of Hematology/Oncology, Department of Medicine, Samsung Medical Center, Sungkyunkwan University School of Medicine, Seoul, Korea; 5 Department of Pathology, Seoul National University Hospital, Seoul, Korea; Sapporo Medical University, JAPAN

## Abstract

Preclinical and clinical studies have suggested that expression of ribonucleotide reductase regulatory subunit M1 (RRM1) and excision repair cross-complementation group 1 (ERCC1) is associated with resistance to gemcitabine and cisplatin, respectively. We evaluated the significance of RRM1 and ERCC1 expression to predict tumor response to gemcitabine plus platinum chemotherapy (GP) and survival in advanced UC. We retrospectively collected tumor samples and reviewed clinical data of 53 patients with unresectable or metastatic UC, who were treated with first-line GP. RRM1 and ERCC1 expression were measured by immunohistochemistry. Among 53 patients, 12 (22.6%) and 26 (49.1%) patients had tumors that demonstrated a high expression for RRM1 and ERCC1, respectively. Twenty-nine (70.7%) of 41 patients with low RRM1 expression achieved a clinical response (complete + partial responses), but only 3 (25.0%) of 12 patients with high RRM1 expression achieved a clinical response after GP (*P*=0.007). Nineteen (70.4%) of 27 patients with low ERCC1 expression achieved a clinical response, while 13 (50.0%) of 26 patients with high ERCC1 expression achieved a clinical response (*P*=0.130). High RRM1 expression was associated with shorter progression free survival and overall survival (PFS *P*=0.006, OS *P*=0.006). Multivariate analysis confirmed that patients with high RRM1 expression had a significantly greater risk of progression and death than those with low RRM1 expression. ERCC1 status was not a significant predictor for PFS and OS. RRM1 expression was predictive and prognostic of clinical outcome in advanced UC treated with gemcitabine plus platinum combination chemotherapy.

## Introduction

Bladder cancer is the seventh most common cancer in men and the seventeenth most common cancer in women in both worldwide [1] and in Korea [2]. Urothelial carcinoma (UC) of the bladder is the most frequent histologic type, accounting for more than 90% of bladder cancers [3] and considered a chemotherapy-sensitive disease. Cisplatin-based combination chemotherapy is the cornerstone of treatment for patients with advanced UC, based on the results of randomized clinical trials [4–8]. One large randomized trial data revealed that efficacy outcomes for gemcitabine plus cisplatin are similar to those seen with MVAC (methotrexate, vinblastine, adriamycin, cisplatin) in terms of objective response, progression-free survival (PFS), and 5-year survival rates [7, 8]. However, given the more favorable toxicity profile of gemcitabine plus cisplatin compared to MVAC, combination chemotherapy with gemcitabine plus cisplatin has recently become preferred over MVAC and has been recognized as an acceptable first-line choice for most patients with advanced UC. Despite of initial high response rates with conventional cisplatin-based chemotherapy, long-term outcomes with current cisplatin-based combination chemotherapy are unsatisfactory.

Ribonucleotide reductase regulatory subunit M1 (RRM1) is the large catalytic subunit of ribonucleotide reductase (RR), the main enzyme catalyzing the conversion of ribonucleoside diphosphates to deoxyribonucleoside diphosphates in the de-novo metabolic pathway of endogenous nucleotides. Gemcitabine, a pyrimidine nucleoside antimetabolite, is a widely used chemotherapeutic agent. The antitumor effect of gemcitabine is mediated by several mechanisms for inhibition of DNA synthesis, including inhibition of RR [9]. Several preclinical and clinical studies have suggested that the RRM1 present in various cancers is associated with resistance to gemcitabine-based chemotherapy [10]. The excision repair cross-complementation group 1 (ERCC1) exerts a crucial role in the nucleotide excision repair (NER) pathway which repairs DNA adducts and other DNA helix-distorting lesions. Because platinum DNA adducts can be removed by the NER pathway, an increase in ERCC1 expression is likely to cause the resistance to cisplatin. Although RRM1 and ERCC1 can be potential biomarkers for resistance to chemotherapeutic agents and patient prognosis, data to correlate RRM1 and ERCC1 status with tumor response to gemcitabine plus platinum in advanced UC are lacking.

Here, we evaluated the significance of RRM1 and ERCC1 to predict tumor response to gemcitabine plus platinum and survival in patients with advanced UC.

## Patients and Methods

Fifty-three patients who were treated with first-line gemcitabine plus platinum chemotherapy for stage IV unresectable or metastatic UC at Seoul National University Hospital between January 2006 and December 2011 were included in this retrospective study. All patients were required to have at least one bidimensionally measurable lesion, Eastern Cooperative Oncology Group (ECOG) performance status (PS) of 0 to 2, and major organs function adequate for chemotherapy. Information on patient demographics, pathologic characteristics, details of treatment, and survival were collected from the medical records. Clinical endpoints in this study were response rates, PFS and overall survival (OS).

Patient records were anonymized and de-identified prior to analysis. This study protocol was reviewed and approved by the Institutional Review Board (IRB) of the Seoul National University Hospital (IRB approval number: H-1208-060-4).

### Treatment and response evaluation

Gemcitabine plus platinum chemotherapy was given for a maximum of 6 cycles or until disease progression. Gemcitabine 1000 mg/m^2^ was administered intravenously on days 1, 8 and 15, and cisplatin 60 mg/m^2^ was given intravenously on day 1 with pre- and post-cisplatin hydration for each 4-week cycle. Patients who had at least one of the following features ─ ECOG PS 2, age older than 75 years, or estimated glomerular filtration rate less than 60 ml/min ─ received carboplatin instead of cisplatin. Carboplatin was administered intravenously on day 1 of each 4-week cycle to achieve a target area under the curve of 5.

Clinical responses were evaluated based on imaging and defined using the Response Evaluation Criteria in Solid Tumors (RECIST) criteria (version 1.1) [11]. PFS was calculated from the date of initial chemotherapy to the date of documented disease progression or death from any cause. OS was calculated from the date of initial chemotherapy to the date of death or the last follow-up visit.

### Tissue microarrays (TMAs) and immunohistochemistry (IHC)

Twenty (37.7%) of 53 tumor specimens had been obtained from cystectomy or nephroureterectomy, 13 tissues (24.5%) from transurethral resection (TUR) of the bladder, and 20 tissues (37.7%) from a core needle biopsy. Thirty-one of 53 tumor specimens were used for TMA construction. The hematoxylin and eosin (H & E stain) slides of all cases were reviewed; an area containing sufficient viable tumor with no hemorrhage or necrosis was selected in each case. One representative core section (2 mm in diameter) was taken from each formalin-fixed paraffin block and embedded in new recipient paraffin blocks (TMA blocks) using a trephine apparatus (Superbiochips Laboratories, Seoula). Remaining 22 cases were not suitable for TMAs construction due to small tumor size, and whole sections were stained in these cases. The immunohistochemical stainings were performed on 4-μm-thick sections taken from TMA slides and whole tissue blocks. All slides were treated to remove wax and rehydrated in a graded series of alcohol solutions. Immunohistochemical staining was performed using the Bond-Max Autostainer (Leica Microsystems, Buffalo Grove, Illinois). Anti-ERCC1 monoclonal antibody (clone 8F1; Neomarkers, Fremont, California) was diluted 1:200. Anti-RRM1 polyclonal antibody (10526-1-AP; ProteinTech Group, Chicago, Illinois), was diluted 1:50. After heat-induced antigen retrieval, primary antibodies were incubated with the samples for 15 minutes. The binding of the primary antibody was detected using the Bond Polymer Refine Detection kit (Leica Microsystems, Buffalo Grove, Illinois) according to the manufacturer’s instructions. Omission of the primary antibody was used as negative control. Formalin-fixed, paraffin-embedded human lung adenocarcinoma and colon adenocarcinoma tissue were used as positive controls for RRM1 and ERCC1, respectively.

### Immunohistochemistry and sample scoring

**Fig 1:**
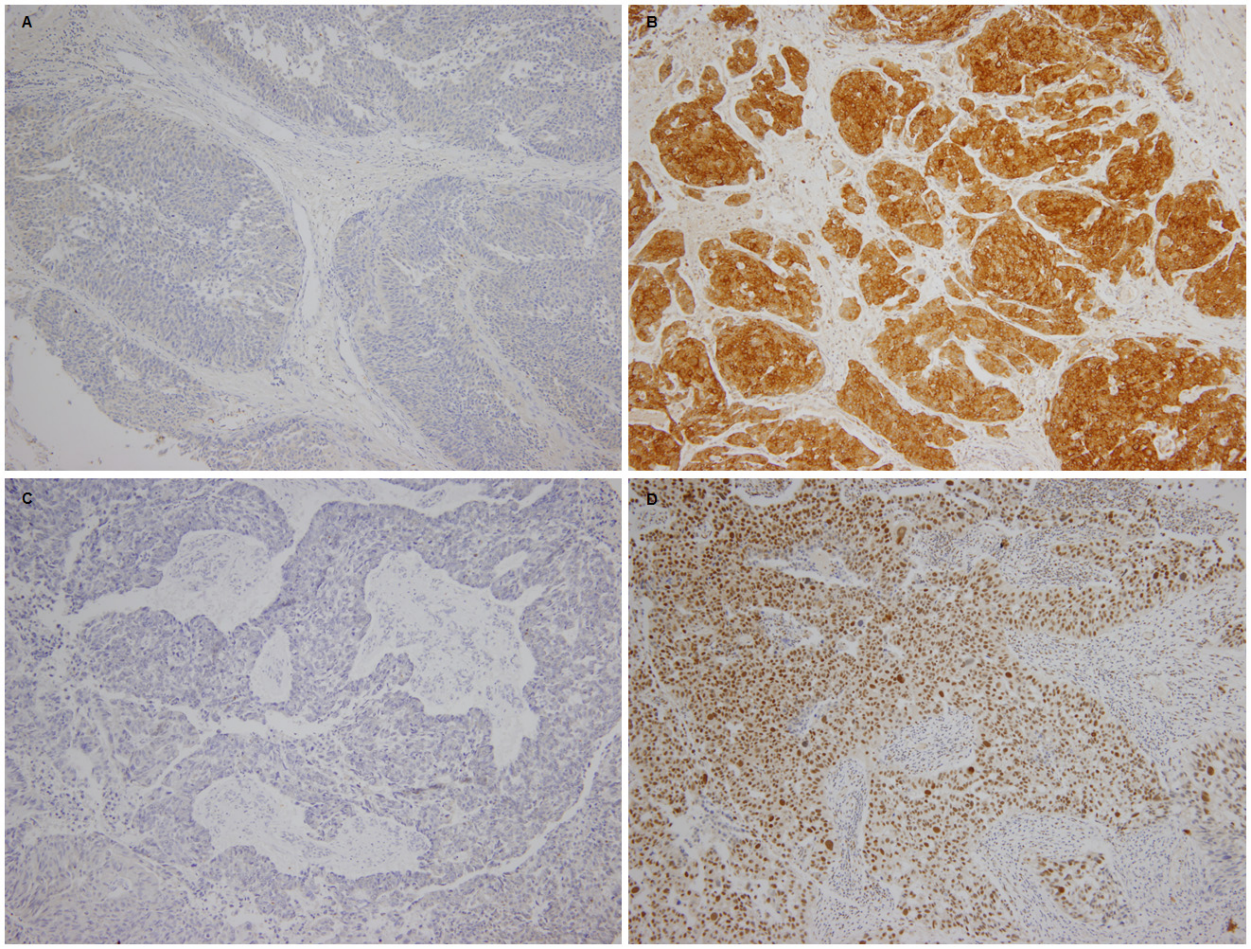
RRM1 and ERCC1 expression in immunohistochemistry (magnified 400×,) (A) Low expression of RRM1, (B) High expression of RRM1, (C) Low expression of ERCC1, (D) High expression of ERCC1, (original magnification, x400 in (A)-(D)).

The staining of whole sections and TMA cores was graded by a single pathologist who was blinded to all clinical data. Fine granular cytoplasmic staining for RRM1 was regarded as positive. The proportions of staining were scored on a scale from 0 to 3 as follows: negative (score 0); focal, 1% to 9% positive (score 1); regional, 10% to 49% positive (score 2); diffuse, ≥50% positive (score 3). The intensity of staining was scored from 0 to 3 (0, absent; 1, weak; 2, moderate; 3, strong). As previously described [12], the semiquantitative H-score for each sample was determined by multiplying the two individual scores and the results divided into high (≥9) and low (<9) expression of RRM1. For ERCC1, the intensity of nuclear staining for ERCC1 was scored as described above. In addition, the percentage of positive tumor nuclei was calculated for each specimen, and a proportion score was assigned (0 if 0%; 0.1 if 1% to 9%; 0.5 if 10% to 49%; and 1.0 if ≥ 50%). This proportion score was multiplied by the staining intensity of nuclei to obtain a final semiquantitative H-score. As previously described [13], tumors with an ERCC1 H-score higher than 1 (i.e., tumors with a staining intensity score of 2 and with 50% or more positive nuclei or with a staining intensity score of 3 and 10% or more positive nuclei) were classified as high expression of ERCC1. Representative immunohistochemical stainings for RRM1 and ERCC1 expression are provided in Fig 1.

### Statistical analysis

Clinicopathologic variables were compared with Pearson chi-square and Fisher exact tests, as appropriate. Univariate and multivariate logistic regression was used to evaluate the relationships between clinical responses and independent variables. The median PFS and OS were calculated using the Kaplan-Meier method. Comparison of survival data was performed using the log rank test. The multivariate analysis of risk factors for survival was made using the Cox proportional hazard model. This analysis was performed using a backward stepwise method. Variables with clinical significance and a significance level of <0.05 were used for covariate entry. Variables with a *P*-value of >0.10 were removed during stepwise analysis. All statistical tests were two-sided, with significance defined as *P* <0.05. All analysis was performed using performed using SPSS, version 19.0 (IBM Corporation, Armonk, New York, USA).

## Results

### Clinicopathologic characteristics

Clinicopathologic characteristics of the study population are summarized in Table 1. The median age of the 53 patients was 66 years (range, 34 to 88years), and 42 patients (79.2%) were male. The primary sites of UC were bladder (60.4%), renal pelvis (28.3%) and ureter (11.3%). Twenty-nine patients (54.7%) had visceral metastases, and 27 patients (50.9%) underwent surgery including radical cystectomy and nephroureterectomy. Among 27 patients who underwent surgery, 18 patients who received curative radical surgery underwent chemotherapy at the time of relapse and the remaining 9 patients with complications such as uncontrolled tumor bleeding received palliative resection of the primary tumor before chemotherapy. Thirty-five (66.0%) patients underwent biopsy or surgical resection of primary tumors; 18 (34.0%) patients underwent biopsy of metastatic tumors.

**Table 1:** Patients’ characteristics according to RRM1 and ERCC1 expression.

		*N* (%)	L-RRM1 *N* = 41(%)	H-RRM1 *N* = 12(%)	*P-*values	L-ERCC1 *N* = 27(%)	H-ERCC1 *N* = 26(%)	*P-*values
Age	≤ 64	23 (43.4%)	18 (78.3)	5 (21.7)	**0.891**	11 (47.8)	12 (52.2)	**0.691**
	> 65	30 (56.6%)	23 (76.7)	7 (23.3)		16 (53.3)	14 (46.7)	
Sex	Male	42 (79.2%)	32 (76.2)	10 (23.8)	**1.000**	22 (52.4)	20 (47.6)	**0.682**
	Female	11 (20.8%)	9 (81.8)	2 (18.2)		5 (45.5)	6 (54.5)	
ECOG Performance status	0–1	42 (79.2%)	33 (78.6)	9 (21.4)	**0.697**	22 (52.4)	20 (47.6)	**0.682**
	>1	11 (20.8%)	8 (72.7)	3 (27.3)		5 (45.5)	6 (54.5)	
Primary origin	Bladder	32 (60.4%)	22 (68.8)	10 (31.3)	**0.160**	17 (53.1)	15 (46.9)	**0.657**
	Renal pelvis	15 (28.3%)	14 (93.3)	1 (6.7)		8 (53.3)	7 (46.7)	
	Ureter	6 (11.3%)	5 (83.3)	1 (16.7)		2 (33.3)	4 (66.7)	
Visceral metastases	Absent	24 (45.3%)	18 (75.0)	6 (25.0)	**0.709**	12 (50.0)	12 (50.0)	**0.901**
	Present	29 (54.7%)	23 (79.3)	6 (20.7)		15 (51.7)	14 (48.3)	
Previous surgery	Yes	27 (50.9%)	25 (92.6)	2 (7.4)	**0.009**	18 (66.7)	9 (33.3)	**0.020**
	No	26 (49.1%)	16 (61.5)	10 (38.5)		9 (34.6)	17 (65.4)	
Chemotherapy regimen	Gem+ Cis	35 (66.0%)	28 (80.0)	7 (20.0)	**0.522**	16 (45.7)	19 (54.3)	**0.288**
	Gem+ Carbo	18 (34.0%)	13 (72.2)	5 (27.8)		11 (61.1)	7 (38.9)	
Biopsy site	Primary	35 (66.0%)	24 (68.6)	11 (31.4)	**0.041**	21 (60.0)	14 (40.0)	**0.066**
	Metastatic	18 (34.0%)	17 (94.4)	1 (5.6)		6 (33.3)	12 (66.7)	

Abbreviations: L-RRM1, low expression of RRM1; H-RRM1, high expression of RRM1; L-ERCC1, low expression of ERCC1; H-ERCC1, high expression of ERCC1; Gem, gemcitabine; Cis, cisplatin; Carbo, carboplatin.

Twelve (22.6%) and 26 (49.1%) patients had tumors that demonstrated a high expression for RRM1 and ERCC1, respectively. Age, sex, ECOG performance status, primary origin of UC, presence of visceral metastases, and chemotherapy regimen were similar between low and high expression of RRM1 as well as ERCC1 expression (Table 1). However, RRM1 and ERCC1 expression trended toward higher expression in patients who had undergone surgery (*P* = 0.009 and *P* = 0.020, respectively). In addition, RRM1 was more highly expressed in primary tumors than metastatic tumors (*P* = 0.041), while ERCC1 was more highly expressed in metastatic tumors than primary tumors (*P* = 0.066).

### Clinical response according to RRM1 and ERCC1 expression

Table 2 shows clinical response rate according to biomarkers. A total of 32 (60.4%) patients achieved a clinical response (CR + PR). Twenty-nine (70.7%) of 41 patients with low RRM1 expression achieved a clinical response after gemcitabine plus platinum chemotherapy as compared to only 3 (25.0%) of 12 patients with high RRM1 expression (*P* = 0.007). Nineteen (70.4%) of 27 patients with low ERCC1 expression achieved a clinical response compared to 13 (50.0%) of 26 patients with high ERCC1 expression (*P* = 0.130). In multivariate analysis, high RRM1 expression was an independent predictor for poorer tumor response to gemcitabine plus platinum chemotherapy (hazard ratio [HR] 7.68, 95% confidence interval [CI] 1.69 to 34.99, *P* = 0.008; Table 3).

**Table 2:** Clinical response assessment according to RRM1 and ERCC1 expression.

Clinical response	L-RRM1 *N* = 41 (%)	H-RRM1 *N* = 12 (%)	*P-*values	L-ERCC1 *N* = 27 (%)	H-ERCC1 *N* = 26 (%)	*P-*values
CR+PR	29 (70.7)	3 (25.0)	**0.007**	19 (70.4)	13 (50.0)	**0.130**
SD+PD	12 (29.3)	9 (75.0)		8 (29.6)	13 (50.0)	

Abbreviations: L-RRM1, low expression of RRM1; H-RRM1, high expression of RRM1; L-ERCC1, low expression of ERCC1; H-ERCC1, high expression of ERCC1; CR, complete remission; PR, partial remission; SD, stable disease; PD, progressive disease.

**Table 3:** Univariate and Multivariate Logistic regression analysis for clinical response.

		Univariate		Multivariate	
Variables		HR (95% CI)	*P-*values	HR (95% CI)	*P-*values
Age	≤ 64	1	0.287	-	-
	> 65	0.55 (0.18–1.67)			
Sex	Female	1	0.804	-	-
	Male	1.19 (0.30–4.70)			
ECOG PS	0–1	1	0.076	1	0.078
	> 1	3.50 (0.88–14.00)		3.82 (0.86–17.01)	
Primary origin	Urinary bladder	1	0.404	-	-
	Renal pelvis	0.41 (0.11–1.57)			
	Ureter	0.57 (0.09–3.55)			
Visceral metastases	Absent	1	0.782	-	-
	Present	0.86 (0.28–2.58)			
Previous surgery	Yes	1	0.041	-	-
	No	3.33 (1.05–10.59)			
Chemotherapy regimen	Gem + Cis	1	0.607	-	-
	Gem + Carbo	1.35 (0.43–4.30)			
RRM1	Low	1	0.008	1	0.008
	High	7.25 (1.67–31.52)		7.68 (1.69–34.99)	
ERCC1	Low	1	0.133	-	-
	High	2.38 (0.77–7.34)			

Abbreviations: HR, hazard ratio; CI, confidence interval; ECOG PS, Eastern Cooperative Oncology Group performance status; Gem, gemcitabine; Cis, cisplatin; Carbo, carboplatin.

### Survival analysis

**Fig 2:**
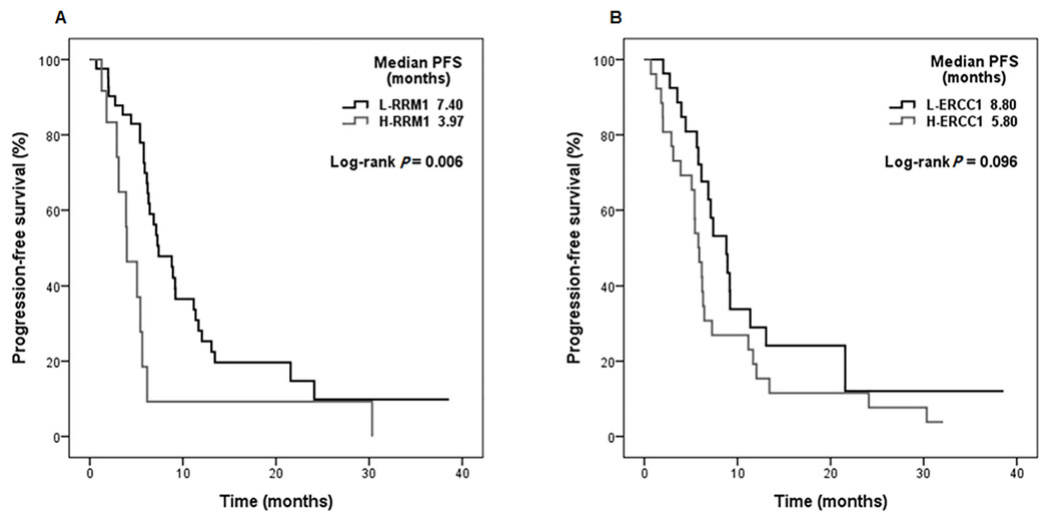
Progression-free survival of patients according to biomarker expression for (A) ribonucleotide reductase subunit M1 (RRM1), (B) excision crosscomplementing gene-1 (ERCC1).

**Fig 3:**
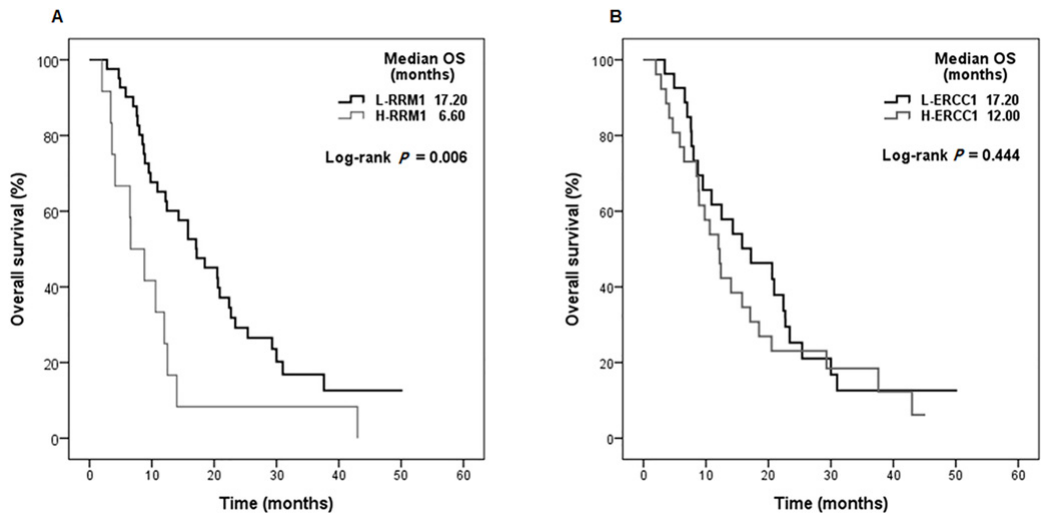
Overall survival of patients according to biomarker expression for (A) ribonucleotide reductase subunit M1 (RRM1), (B) excision crosscomplementing gene-1 (ERCC1).

The median follow-up from initiation of chemotherapy was 12.5 months (range 2.0–50.2months). The median PFS and OS were 6.4 months and 14.0 months, respectively. PFS and OS were significantly shorter for patients with high RRM1 expression than for those with low RRM1 expression (PFS median 3.97 months vs. 7.40 months, *P* = 0.006; Fig 2A, OS median 6.60 months vs. 17.20 months, *P* = 0.006; Fig 3A). ERCC1 status was not statistically significant in terms of PFS and OS (PFS *P* = 0.096, OS *P* = 0.444; Figs 2B and 3B).

Table 4 shows PFS and OS results for the univariate and multivariate Cox regression analyses. Multivariate analysis confirmed that high RRM1 expression (HR 2.41, 95% CI 1.02 to 5.72, *P* = 0.046), poor performance status (HR 2.21, 95% CI 1.02 to 4.81, *P* = 0.045), and visceral metastases (HR 2.32, 95% CI 1.20 to 4.49, *P* = 0.012) were significant risk factors for PFS. Significant risk factors for OS were High RRM1 expression (HR 3.21, 95% CI 1.49 to 6.92, *P* = 0.003), male gender (HR 3.15, 95% CI 1.27 to 7.76, *P* = 0.013), poor performance status (HR 2.62, 95% CI 1.18 to 5.81, *P* = 0.018), and visceral metastases (HR 1.96, 95% CI 1.01 to 3.77, *P* = 0.045).

**Table 4:** Univariate and Multivariate Cox regression analyses for PFS and OS.

Variables		Progression-free survival				Overall survival			
		Univariate		Multivariate		Univariate		Multivariate	
		HR (95% CI)	*P-*values	HR (95% CI)	*P-*values	HR (95% CI)	*P-*values	HR (95% CI)	*P-*values
**Age**	≤ 64	1	0.141	-	-	1	0.894	-	-
	> 65	0.63 (0.34–1.17)				0.96 (0.53–1.76)			
**Sex**	Female	1	0.074	1	0.051	1	0.044	1	0.013
	Male	0.50 (0.23–1.07)		2.60 (1.00–6.78)		2.25 (1.02–4.97)		3.15 (1.27–7.76)	
**ECOG PS**	0–1	1	0.045	1	0.045	1	0.007	1	0.018
	> 1	2.39 (1.13–5.05)		2.21 (1.02–4.81)		2.72 (1.32–5.63)		2.62 (1.18–5.81)	
**Primary origin**	Urinary bladder	1	0.120	-	-	1	0.185	-	-
	Renal pelvis	0.63 (0.32–1.24)				0.57 (0.28–1.14)			
	Ureter	0.33 (0.10–1.11)				0.54 (0.19–1.53)			
**Visceral metastases**	Absent	1	0.040	1	0.012	1	0.251	1	0.045
	Present	1.92 (1.03–3.58)		2.32 (1.20–4.49)		1.43 (0.78–2.61)		1.96 (1.01–3.77)	
**Previous surgery**	Yes	1	0.202	-	-	1	0.141	-	-
	No	1.48 (0.81–2.72)				1.56 (0.86–2.82)			
**Chemotherapy regimen**	Gem+Cis	1	0.034	1	0.069	1	0.213	-	-
	Gem+Carbo	2.01 (1.05–3.83)		2.13 (0.94–4.79)		1.48 (0.80–2.76)			
**RRM1**	Low	1	0.008	1	0.046	1	0.008	1	0.003
	High	2.64 (1.29–5.38)		2.41 (1.02–5.72)		2.52 (1.28–4.98)		3.21 (1.49–6.92)	
**ERCC1**	Low	1	0.100	1	0.061	1	0.445	-	-
	High	1.67(0.91–3.07)		2.14 (0.97–4.75)		1.26 (0.70–2.26)			

## Discussion

In recent years, a number of studies have evaluated biomarkers as predictive and/or prognostic markers in various tumors. However, in the field of cytotoxic agents, there has been no routinely used biomarker for predicting treatment response. Although we cannot depend on a single biomarker to determine optimal chemotherapy for individual patients in current practice setting, using a set of biomarkers validated in large-scale prospective studies would be usefulness in guiding therapeutic decision making and improving ultimately treatment outcome.

Our study included 53 patients and evaluated two biomarkers, RRM1 and ERCC1, for the assessment of the clinical efficacy of gemcitabine plus platinum combination chemotherapy in patients with advanced UC. RRM1 expression by IHC was an independent unfavorable predictive and prognostic factor in this cohort. In addition, data from a multivariate Cox analysis were consistent with previous results—that the presence of visceral metastases and ECOG performance score > 1 predict poor outcomes with chemotherapy [14]. However, we were unable to demonstrate a statistically significant interaction between ERCC1 expression and treatment outcomes for all patients.

Although the majority of the previous studies regarding RRM1 have been conducted in non-small cell lung cancer (NSCLC), there have been some conflicting results about the predictive value of RRM1. A recent meta-analysis of 18 studies evaluating a predictive role of RRM1 expression by IHC or quantitative real-time polymerase chain reaction (qRT-PCR) in the efficacy of gemcitabine-based regimens in patients with advanced NSCLC showed that low or negative RRM1 expression in advanced NSCLC was associated with higher response rate to gemcitabine-based regimens and a better prognosis [12]. In UC, several studies have been conducted to evaluate the predictive or prognostic value of RRM1 in UC. Previously, Harshman et al. [15] reported that high RRM1 expression analyzed by immunofluorescence combined with automated quantitative analysis (AQUA) may be prognostic for improved survival in patients aged < 70 years with muscle-invasive UC. On the other hand, Bellmun et al. [16] failed to demonstrate the predictive value of RRM1 mRNA levels for survival outcome as well as chemotherapy response in advanced bladder cancer in patients receiving cisplatin-based chemotherapy, but not limited to combination with gemcitabine. These conflicting data may result from differences in study population and methods for evaluating biomarkers. Similar with our results, Shilkrut et al. [17] recently reported that the expression of RRM1, but not ERCC1, may predict response to gemcitabine-based chemoradiotherapy and worse cancer-specific survival in patients with muscle-invasive UC. They suggested that low RRM1 expression may help identify patients suitable for gemcitabine-based chemoradiotherapy.

ERCC1 as a biomarker of treatment efficacy or survival has been studied in many solid tumors. A number of studies in NSCLC patients suggested that ERCC1 was a predictive or a prognostic marker, while some other studies showed no correlation between ERCC1 expression and tumor response or survival [13, 18–21]. In bladder cancer, several studies have shown that ERCC1 can be a potential prognostic and/or biomarker of the efficacy of platinum-based chemotherapy [16, 22, 23]. However, recently Brambilla and Soria did not observe the predictive effect of immunostaining for ERCC1 protein in NSCLC [24]. They also demonstrated that currently available antibodies did not detect the unique functional isoform of ERCC1 that had full capacities for NER and platinum resistance. Technical issues such as clinical reproducibility or specificity of ERCC1 antibodies need to be overcome before implementation in clinical practice.

While upper urinary tract UC shares many features with bladder UC, distinctive clinico-patholgical characteristics of upper urinary tract UC are observed in several studies [25, 26]. In this study, there were no significant differences in the high RRM1 and ERCC1 expression rates between bladder UC and upper urinary tract UC. Our results showed a significant association between RRM1 expression and sites of biopsy; in other words, there was higher RRM1 expression in primary sites than in metastatic sites. And there was a trend of lower ERCC1 expression in primary sites than metastatic sites. Thus, a subanalysis was performed for the 25 patients who underwent a biopsy from the primary site. The predictive value of RRM1 and ERCC1 did not change from the results of the original 53 patients following this subanalysis (data not shown). Expression of RRM1 and ERCC1 might change due to selective events in the metastatic process, or intratumoral heterogeneity might cause different RRM1 and ERCC1 expression between primary and metastatic sites.

Our study has some limitations. This was a retrospective single-center study conducted in a relatively small sample size. In addition, there might be bias from the sample preparation and intratumoral biomarker heterogeneity, although we selected and read the most representative tumor areas. Therefore, our results should be interpreted cautiously and further prospective studies with a larger sample size are definitely. Nevertheless, this study is clinically meaningful because the results provide evidence of the clinical implications of RRM1 protein expression by IHC in patients with advanced UC and suggests the direction of future prospective clinical trials.

In conclusion, our data support that RRM1 protein expression by IHC may provide additional information regarding clinical outcomes after first-line gemcitabine plus platinum chemotherapy and can help develop appropriate treatment plans in patients with advanced UC.
